# An ethic of care? Academic administration and pandemic policy

**DOI:** 10.1177/1473325020973386

**Published:** 2021-03

**Authors:** Stephanie A Bryson

**Affiliations:** School of Social Work, Portland State University, Portland, OR, USA

**Keywords:** Auto-ethnography, reflexivity, social work education, social justice

## Abstract

This reflexive essay examines the adoption of an intentional ‘ethic of care’ by social work administrators in a large social work school located in the Pacific Northwest. An ethic of care foregrounds networks of human interdependence that collapse the public/private divide. Moreover, rooted in the political theory of recognition, a care ethic responds to crisis by attending to individuals’ uniqueness and ‘whole particularity.’ Foremost, it rejects indifference. Through the personal recollections of one academic administrator, the impact of rejecting indifference in spring term 2020 is described. The essay concludes by linking the rejection of indifference to the national political landscape.

## My first zoom meeting

“Oh my gosh, your cat is huge!”

“This is my son, Charlie. He’s five. He’s so happy to see other people’s kids!”

“Watch this: I can put my chicken to sleep on her back.”

Pets and children were the keys to the kingdom in the first all-program Zoom meeting I held on March 19, 2020, a Thursday night, from 7-8 pm.

I had billed it as a *Virtual Connection Space* and literally plunked down the names, in my Google calendar, of all 400 BSW students, advisors, inclusion coordinators, administrative staff, full-time faculty, and adjunct faculty. In the Google calendar invite, I aimed for the language of “holding space” often used by my millennial activist colleagues:

This is not a mandatory meeting, just a space for BSW students and faculty to connect, ask questions about spring term, and see each other's children and pets. Do not worry about replying. Just drop in for 5 minutes if you feel like it and want to say hi.

As the time grew near, my chest grew tight. I repositioned my laptop for a better jawline angle. I had no idea if it would work, how it would work, what I was doing. In four years as BSW director, I had taught, hired five faculty, and conducted Director Dialogues with 60 students at a time, but I had done nothing like this.

More than fifty people showed up. The fear was palpable. A vocal trans activist admitted in soft lamplight that their social anxiety and OCD were “super activated by Corona Virus.” One after another, BSW students chimed in to report feeling worried, scared, and vulnerable. Some asked questions, which I answered based on emerging data and twice weekly meetings with upper administrators. Voices broke or remained quiet. Mothers who were home with kids looked exhausted. A Hybrid student suppressed sobs. Her husband, a grocery store manager, was pulling double shifts. She was at home with two toddlers and a baby. Even typically unflappable faculty seemed tentative, vulnerable, and eager to connect that night in March.

Initially, some people remained off camera, black boxes with names I recognized and names I did not. Eager to hear from everyone who might want the chance to speak (I’ve learned many things about trauma informed Zoom since then), I invited people to just be in the space, however they were, no matter what their houses and lives looked like. Cameras clicked on to reveal kids cuddling with their parents on couches, the torsos of parents trying to cook dinner, faces lit by dashboards on the long commute home. One by one, in the strange comfort of this digital space, students introduced kids, partners, cats, dogs, chickens, and goats. They talked about the jobs they lost or were losing. They talked about the overnight shifts they were willing to pick up. They asked for sobriety support. A student still doing outreach shifts asked for PPE.

It was no bonfire, but in the eerie darkness of March, with Covid cases mounting by the day in nearby Washington, this moment was a spark of joy-in-connection, fed by a deliberate ethic of care.

## An ethic of care

I’ve always admired thinkers and writers who can tell a story in a clear, straight line. Olena Hankivsky is one of these thinkers. A Canadian policy scholar, Hankivsky (2004) writes about what makes North American social policy uniquely unable to respond to crises of care like Covid-19. The answer is liberalism (which might be hard to recognize in its present guise as Neoliberal Apocalypse, but trust her, it’s still liberalism).

Specifically, Hankivsky describes two features of liberalism that make it especially bad at caring for people in need. The first feature is an enduring public/private split in which all human need is relegated to a gendered and racialized *private sphere*, while *public need* is simultaneously stigmatized. The second characteristic of liberalism that undermines responsiveness to need is the presumption of a *universal citizen*, with an accompanying bureaucratic indifference to the specific and historical valence of peoples’ lived experience. In presuming an autonomous, self-interested citizen willing to extend political entitlements to other independent, self-sufficient subjects—but only if it deprives them little liberty—liberalism presumes a normative (cishetwhitemale) citizen. It projects onto non-normative individuals blame for the structural conditions afflicting them. Pre Covid-19, you could observe this phenomenon at play in the U.S. school-to-prison pipeline, in the punitive and sexist welfare state, and in racist policing and prison systems. During Covid-19, it has become shockingly clear how little some U.S. policymakers care about care, prizing economic recovery over all else. In Hankivsky’s (2004) words, under liberalism, “Care has … seemed irrelevant to public life (p. 6).”

The antidote is an *ethic of care*. As Hankivsky (2004) describes it, an ethic of care “privileges networks of human interdependence that challenge the public/private divide and the concomitant role that care plays in such relations” (p. 2). Rooted in the political theory of recognition (c.f., Fraser, 1997, 2000, 2001; Fraser and Honneth, 2003), a care ethic responds to crisis by attending to individuals’ uniqueness and ‘whole particularity.’ Foremost, it rejects indifference.

## Rejecting indifference

If we—as a program, a school, and for the most part, an institution of higher education—got one thing right in spring term 2020, it was this simple act of *rejecting indifference*.

On March 19, 2020, the day I held the first of six large Zoom gatherings, I lay in bed listening to the increasingly ragged yelps and shrieks of my 12-year autistic daughter—a telltale sign of her dawning realization that everything was gone. Gone: all the routine that anchors her life and makes the world intelligible. Gone: the school bus, her classroom, her teacher, her classmates. Gone: the duck pond, the mall, shopping, stores, PT, OT, speech, her nanny. After ten 10-hour days spent in Zoom meetings on a beige faux suede couch trying to mute the agitated screams of my child, I found myself overwhelmed and depressed. Scrolling through my *NY Times* feed, I landed on an article about burnout among medical professionals (Grant, 2020). I clung to and exhorted these three heuristics for the rest of the term: 1) Listening with empathy increases a sense of control; 2) Adjusting job demands by lowering expectations is critical; and 3) Asking for and receiving help is essential for *everyone—including students, faculty, staff, and administrators.* In my first Zoom meeting, I practiced these things. They seemed to work. I practiced them in faculty meetings. They seemed to work. I practiced them in program director meetings. Then I disseminated them widely in the BSW program.

Between March 2 and June 11, I wrote 25 emails to BSW students and faculty, first twice weekly—when we were moving to Stay-at-Home orders and adjusting to remote teaching—and then weekly until graduation. While this might seem a wholly unremarkable act, this practice/policy was a radical departure from business-as usual and a concrete example of rejecting indifference. I did not always want to write the emails. I did not always have wisdom to share, or frankly, a grasp on my own mental health. I pushed myself to communicate more often, with more honesty, and with more warmth than in typical times. I settled into a routine in which I worked all week to gather the best and most actionable data from the university, the best financial resources, and the week’s best journalism , and then I sent this compilation late on Friday, a kind of weekend love letter to the entire BSW program.

**Figure 1.**
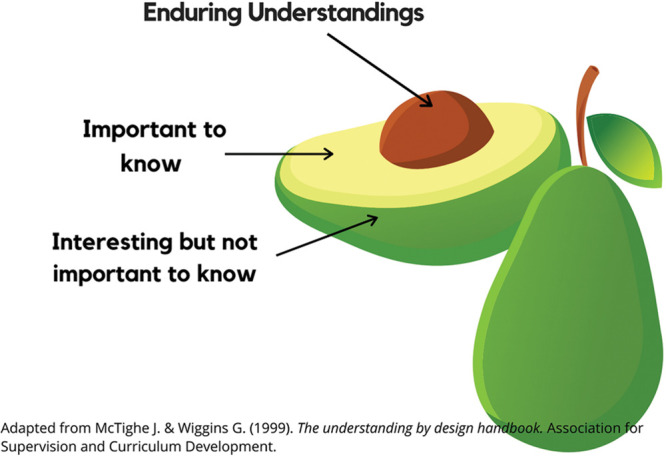
Avocado Model of Enduring Understandings

Midway through the term, when I worried we would lose students because of widespread anomie, I invited them into a pedagogical conversation about “enduring understandings” (see Figure 1) to help them reframe their educational experience and hang on.

If you find that you can't concentrate or write very well or if you are worried that you are missing out on important information in your classes, I want to share a useful concept: enduring understandings. What you will remember from this time years from now is the pit of the avocado. Even if you can take in very little new information in your classes, your avocado pit will be made up of this entire experience, and it all counts toward your education. We are all learning, no matter what. This concept may also help you to manage your workload: What one thing can you take away from your classes that helps you to think about and plan for your future?

I started to send Empathic Cat Videos, which were videos meant to capture the ennui and demotivation of the moment. I narrated one, badly, but students continued to request them. I began to include other video moments of joy (the cast and friends of Hairspray singing from 200 living rooms). I emailed BSW faculty and students together in the same email. This simple act rejected indifference by collapsing the split we often erect between faculty and students. It brought us all together in community. It acknowledged our shared experience and our shared needs. And it kept the communication lines open so students could reach out to me or to other faculty.

When student needs became known—they were unemployed but not receiving unemployment benefits, they were food insecure, they were denied CARES Act funding because they had been enrolled exclusively in online classes for 2019-2020—program directors reported this to our Deans, who then took concerns to the Provost and the President. We centered students, particularly those from Black, Indigenous, and People of Color (BIPOC) communities—as we said we would in a racial equity curriculum commitment we adopted in 2016.

In the four years before the pandemic, I spent considerable time running interference between the university’s inevitable push for increased enrollment and the quality of the experience for students, faculty, and staff. During spring term of the pandemic, we became advocates for students. We drove the agenda of upper administration by leveraging student retention, the coin of the realm. We held and comforted faculty—especially those who lost loved ones to Covid. We sought to appreciate their labor regularly and to remind them to ask for help. Our online and hybrid coordinators stepped up to teach us all Zoom. And program directors met twice weekly with our Dean and Associate Dean, which proved an invaluable sounding board for decision-making and support for our unique roles.

At the university level, miraculous and entirely unprecedented things happened. On March 31, the university made classes option Pass/No Pass. It refunded student fees for all services impacted by the Stay-at-Home order. When Student Financial Services announced that it would not charge late fees on tuition, and when Parking Services stopped charging and ticketing, I knew we had entered a new era of *rejecting indifference*.

## Reflection and reverie

What worked in the first term of the pandemic, for an access institution in the Pacific Northwest where some of the first cases of Covid-19 were diagnosed, was treating our students, and each other, like members of a beloved community. What worked was removing the stigma attached to need. What worked was rejecting bureaucratic indifference. What worked was trusting each other.

What worked was setting fire to the flimsy and fictitious barrier dividing the public from the private, faculty from student, faculty from administration. What worked was refusing to cleave grief from celebration. We grieved George Floyd; we celebrated BIPOC students graduating. We grieved the loss of 2020; we celebrated its strange new intimacies. We grieved the callousness of our nation’s narcissism; we celebrated wave upon wave of protest, refusal, and resistance.

As I write, I am certain these thoughts will be washed away like names written in sand. As I write, 17-year old vigilantes are celebrated in social media for shooting BLM protesters, while 6.3 million people have tested positive for Covid-19 and 190,000 have died in the U.S. alone. As I write, I dream of a nation that responds, perhaps over a number of generations, to the lessons wrought by 2020; that wakes from its brush with tyranny; that moves courageously toward reconciliation and reparations; that recognizes the specific, historical harms produced by colonization, slavery, and white supremacy.

I dream of a nation that realizes that sedimented inequality spells the end to all futures, not just those deemed expendable. Our survival as a species, it seems, rests on our ability to *see* each other, to *care* about what we find there, and to *refuse* the easy path of *individual liberty through indifference.*
